# Disodium Cromoglycate Reverses Colonic Visceral Hypersensitivity and Influences Colonic Ion Transport in a Stress-Sensitive Rat Strain

**DOI:** 10.1371/journal.pone.0084718

**Published:** 2013-12-18

**Authors:** Siobhan Yvonne Carroll, Siobhain Mary O’Mahony, Susan Grenham, John Francis Cryan, Niall Patrick Hyland

**Affiliations:** 1 Alimentary Pharmabiotic Centre, University College Cork, Cork, Ireland; 2 Department of Pharmacology & Therapeutics, University College Cork, Cork, Ireland; 3 Department of Anatomy & Neuroscience, University College Cork, Cork, Ireland; University of California, Los Angeles, United States of America

## Abstract

The interface between psychiatry and stress-related gastrointestinal disorders (GI), such as irritable bowel syndrome (IBS), is well established, with anxiety and depression the most frequently occurring comorbid conditions. Moreover, stress-sensitive Wistar Kyoto (WKY) rats, which display anxiety- and depressive-like behaviors, exhibit GI disturbances akin to those observed in stress-related GI disorders. Additionally, there is mounting preclinical and clinical evidence implicating mast cells as significant contributors to the development of abdominal visceral pain in IBS. In this study we examined the effects of the rat connective tissue mast cell (CTMC) stabiliser, disodium cromoglycate (DSCG) on visceral hypersensitivity and colonic ion transport, and examined both colonic and peritoneal mast cells from stress-sensitive WKY rats. DSCG significantly decreased abdominal pain behaviors induced by colorectal distension in WKY animals independent of a reduction in colonic rat mast cell mediator release. We further demonstrated that mast cell-stimulated colonic ion transport was sensitive to inhibition by the mast cell stabiliser DSCG, an effect only observed in stress-sensitive rats. Moreover, CTMC-like mast cells were significantly increased in the colonic submucosa of WKY animals, and we observed a significant increase in the proportion of intermediate, or immature, peritoneal mast cells relative to control animals. Collectively our data further support a role for mast cells in the pathogenesis of stress-related GI disorders.

## Introduction

Abdominal visceral pain and altered bowel habits are cardinal symptoms of the stress-related gastrointestinal (GI) disorder, irritable bowel syndrome (IBS) [1,2]. Moreover, the interface between psychiatry and IBS is well established, with anxiety and depressive disorders the most frequently occurring comorbid conditions [3]. It is therefore not surprising that rodent models of anxiety and depression, such as the Wistar Kyoto (WKY) rat [4], display GI features akin to those associated with IBS, for example increased abdominal visceral pain [5-7], reduced colonic accommodation [8] and altered colonic fluid and electrolyte transport [9,10], all key pathophysiological features which support the use of the WKY rat as a valid pre-clinical paradigm for examining stress-related GI disorders, and in particular IBS [11].

The evidence to date implicating mast cells in the pathogenesis and pathophysiology of IBS [12-15], and the contribution of their mediators to the development of visceral hypersensitivity [12,13,16,17] is robust, and supports the targeting of mast cells in the management of stress-related GI disorders. However, not all preclinical models of visceral hypersensitivity display significantly increased colonic mucosal mast cells (MMC) [18-20], and we have previously observed a distinct increase in colonic submucosal, connective tissue-like, mast cells following early life stress [18]. Moreover, the effects of the mast cell stabiliser, doxantrazole on abdominal pain have yielded conflicting results in rat models of visceral hypersensitivity [18,19,21,22]. However, the selectivity of mast cell stabilisers for specific mast cell populations has been exploited to confirm mast cell heterogeneity with respect to mast cell mediator release [23], and to discriminate the contribution of MMC from connective tissue mast cells (CTMC) on GI function in the rat [24,25]. Therefore, in this study we have examined the effects of the CTMC stabiliser [26], disodium cromoglycate (DSCG) on abdominal visceral pain in a stress-sensitive rat strain. Additionally, given the association between stress-related GI disorders and altered bowel habit, we sought to examine the effects of DSCG on colonic secretory function in anxiety-prone WKY rats. 

## Materials and Methods

### Ethics statement

All animal experimentation was approved by the University College Cork Animal Experimentation Ethics Committee and conducted in accordance with the European Community Council directive (86/69/EEC). 

### Animals

Male weight-matched anxiety-prone WKY and normo-anxious Sprague Dawley rats (SD; 250-300g) were purchased from Harlan U.K. (Bicester, Oxon, United Kingdom). Animals were maintained under standard controlled conditions with a 12 hour light/dark cycle (lights on 7.00am). Rats were allowed to acclimatise for one week prior to experimentation, were briefly handled daily, though were not habituated to the colorectal distension (CRD) environment. Food and water were available *ad libitum*, with the exception of those animals used for CRD, which were fasted overnight before experimentation.

### Colorectal distension

CRD was conducted as previously described in detail by our group [27]. Rats were briefly anaesthetised with isoflurane and a latex balloon (6 cm in length) was inserted into the descending colon 1 cm from the anus. Animals were allowed to recover for 10 minutes before undergoing CRD. The balloon was connected to a barostat controlled by a computer using Protocol plus TM distension software (G&J electronic, Toronto, Ontario, Canada), and was inflated from 0-80 mm Hg over 8 minutes. During this time, the pressure at which the first pain behaviour was observed (threshold), and total number of visceral pain behaviours were visually recorded by an observer blinded to the experimental groups. Given the short half-life of DSCG [28] animals were treated 24, 6 and 1 hour prior to CRD with vehicle (saline), 25 mg kg^-1^ or 50 mg kg^-1^ DSCG intraperitoneally (i.p.). Previous studies have used a similar pre-treatment regimen to stabilise rat submucosal mast cells [29], and doses of DSCG in the range of 10 mg kg^-1^ to 50 mg kg^-1^ i.p. have previously been demonstrated to decrease nerve damage-induced hyperalgesia [29], inhibit mast cell activation [30] and protect against gastroduodenal ulcers [31] in rats. 

### Assessment of colonic mast cell inhibition

To determine whether *in vivo* administration of DSCG stabilised colonic mast cells, following CRD animals were euthanized by carbon dioxide (CO_2_) inhalation and distal colon samples (1cm proximal to the anus) were collected for analysis of RMCPII and histamine. Briefly, 3 cm segments of distal colon were rapidly immersed in 2 ml Hanks balance salt solution (HBSS) in 5 ml tubes oxygenated with carbogen gas (95% O_2_/5% CO_2_), maintained at 37°C and stimulated with compound 48/80 (10 μg ml^-1^; Sigma-Aldrich) for 30 minutes in a shaking water bath. Aliquots of stimulated colon supernatants were frozen at -80°C prior to assessment of rat mast cell protease II (RMCPII; Moredun Scientific Limited) or histamine (Labor Diagnostika Nord GmbH & Co, KG, Nordhorn, Germany) release by enzyme-linked immunosorbent assay kits according to the manufacturer’s instructions. 

### Colonic ion transport

In a separate group of WKY and SD animals we assessed the sensitivity of short circuit current (Isc) to compound 48/80 (10 µg ml^-1^) and DSCG (10^-3^M) *in vitro*. Animals were euthanized by CO_2_ inhalation, after which descending colon samples (collected up to 6 cm proximal to the rectum) were obtained, carefully rinsed of any faecal matter and the circular and longitudinal muscle layers, along with the myenteric plexus, removed by blunt dissection. Colon segments were then placed in NaviCyte vertical Ussing chambers (Harvard Apparatus, Kent, United Kingdom), and each side of the tissue was bathed in 5 ml of circulating oxygenated (95% O_2_/5% CO_2_) Krebs buffer (1.2 mM NaH_2_PO_4 ,_ 117mM NaCl, 4.8mM KCl, 1.2mM MgCl_2_, 25mM NaHCO_3,_ 11mM CaCl_2_, 10mM glucose) maintained at 37°C. Tissues were then voltage clamped at 0 mV (EVC-4000 or DVC-1000; World Precision Instruments, Sarasota, Florida, United States of America) and short-circuit current (Isc) digitally recorded (LabTrax 4/16; World Precision Instruments) and analysed using DataTrax 2 software (World Precision Instruments). Tissues were allowed to stabilise for 30 minutes after which either vehicle or DSCG were added (30 minutes), followed by either vehicle or compound 48/80 for 1 hour. Bethanechol (100 μM; Sigma-Aldrich) and forskolin (10 μM; Sigma-Aldrich) were added at the end of each experiment. All drug additions were to the basolateral half of the Ussing chamber. Tissues were not paired, and WKY and SD preparations were usually carried out on different days. Results were converted to μA.cm^-2^.

### Immunohistochemical and histochemical identification of submucosal mast cells from Wistar Kyoto rats

Descending colon samples were collected from experimentally naïve animals and were fixed in 4% paraformaldehyde, cryo-preserved in 30% sucrose and subsequently cryostat sectioned at 12 μm. For histological identification of mast cells, Alcian blue (Sigma-Aldrich, Poole, United Kingdom) and safranin (Sigma-Aldrich) staining was conducted as described by others [17], and previously used by our group to identify submucosal mast cells in rat colon [18]. Briefly, slides were rinsed for 1 minute in 10 mM PBS, and then incubated for 1 minute in 0.125N hydrochloric acid (HCL). Slides were then incubated for 1 hour with 1% Alcian blue in 0.7N HCL, for 1 minute in 0.125N HCL followed by 2 minutes incubation with 0.5% safranin in 0.125N HCL. Slides were subsequently dehydrated and mounted with DPX mounting media. For immunohistochemical staining of RMCP II, sections were incubated overnight in a humidified chamber at 4°C with sheep anti-rat RMCPII (1:500; Moredun Scientific Limited, Midlothian, Scotland), after which sections were incubated with a cyanine-conjugated secondary antibody (1:100; Jackson ImmunoResearch Laboratory Inc, Suffolk, United Kingdom) for two hours at room temperature. Slides were finally washed and mounted with bicarbonate buffered glycerol and stored at 4°C.

### Histochemical identification of peritoneal mast cells from Wistar Kyoto rats

To isolate peritoneal mast cells, animals were first euthanized by CO_2_ inhalation after which 20 ml of sterile physiological saline was injected into the peritoneal cavity and the area massaged for 5 minutes. Peritoneal exudates were collected and filtered through a 70 µm cell strainer, centrifuged at 1200 rpm for 5 minutes and re-suspended in 1 ml of sterile physiological saline. Mast cells were then purified by Percoll density gradient as previously described [32]. The purified mast cells were then cytocentrifuged at 350 x g for 5 minutes onto glass slides and were left to dry overnight before fixation in Carnoy’s fixative. Mast cells were then stained with Alcian blue and safranin as outlined above. 

### Mast cell quantification

Colonic submucosal mast cells were counted and classified as Alcian blue, safranin or RMCPII positive. For submucosal cell counts, cells present in 3 sections, randomly selected from 3 slides per animal (n=9-15 animals) were quantified, and were subsequently expressed as total number of mast cells per section given the low numbers of cells observed. For quantification of RMCPII-containing MMC, sections and slides were similarly selected, however given the significantly greater numbers of MMC per section, the number of RMCPII immuno-positive cells per mm^2^ were quantified in a similar manner to that previously reported by our laboratory [18]. Peritoneal mast cells were classified based on their Alcian blue or safranin staining characteristics, and were expressed as a percentage of total mast cells per slide. 

### Statistical analysis

Data are expressed as mean ^+^/_-_ standard error of the mean throughout. *p*<0.05 was considered significant. Colonic mast cell counts were analysed using an unpaired one-tailed Student’s t-test and peritoneal mast cells were analysed using a two-way analysis of variance (ANOVA) followed by LSD post hoc test. Threshold and total number of pain behaviours between WKY and SD tissues were analysed using a Student’s t-test. Comparisons between vehicle, 25 mg kg^-1^ or 50 mg kg^-1^ DSCG on threshold, total number of pain behaviours and release of RMCPII and histamine were analysed using one-way ANOVA followed by Bonferroni’s multiple comparison post-test. Changes in Isc in response to compound 48/80 and DSCG were analysed using two-way ANOVA followed by LSD post hoc test. All statistical analysis was performed using SPSS 14.0 for Windows.

## Results

### Disodium cromoglycate inhibits visceral hypersensitivity in stress-sensitive Wistar Kyoto rats

**Figure 1:**
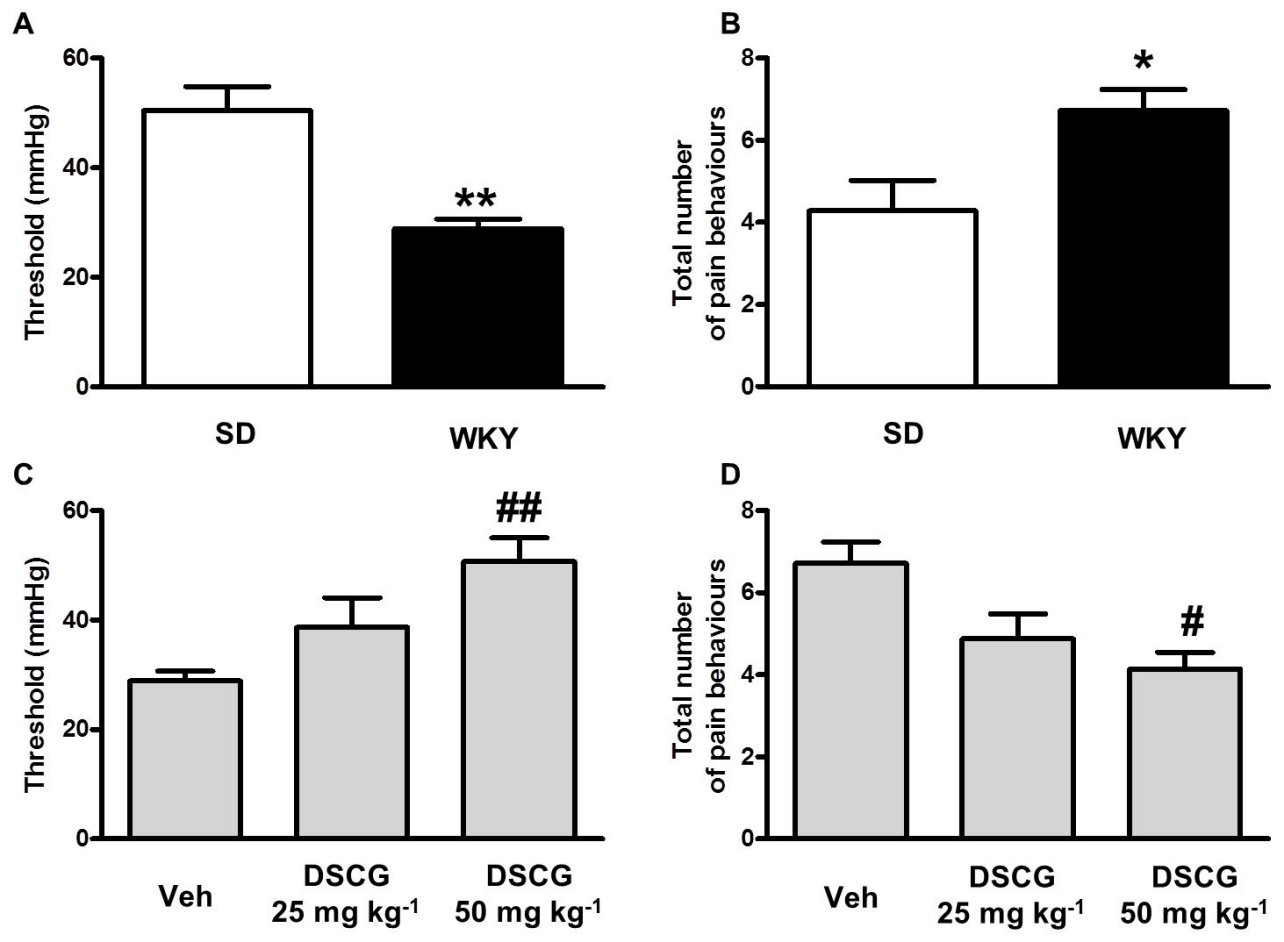
Disodium cromoglycate inhibits visceral hypersensitivity in stress-prone Wistar Kyoto rats. Colorectal distension-induced pain behaviors in Wistar Kyoto (WKY) animals were significantly greater than those observed in Sprague Dawley (SD) animals (A, threshold; B, total pain behaviours). Pre-treatment with 50 mg kg^-1^ disodium cromoglycate (DSCG) significantly increased the pressure required to induce the first pain behaviour (C, threshold) and decreased the total number of pain behaviours (D) in WKY animals. n= 7-8. * *p*<0.05, ** *p* <0.01, *versus* SD. # *p* <0.05, ## *p* <0.01, *versus* vehicle.

WKY rats displayed a viscerally hypersensitive phenotype relative to SD animals (Figure 1 A and B), and this was sensitive, in a dose-dependent manner, to pre-treatment with DSCG (F(2, 23)=12.5, *p*<0.001 (threshold); (F(2, 21)=5.99, *p*<0.05 (total pain behaviours)), where 50mg kg^-1^ DSCG significantly increased threshold (*p*<0.01; Figure 1C) and significantly decreased total pain behaviours (*p*<0.05; Figure 1D). 

### Colonic release mast cell mediators is insensitive to inhibition by disodium cromoglycate

**Figure 2:**
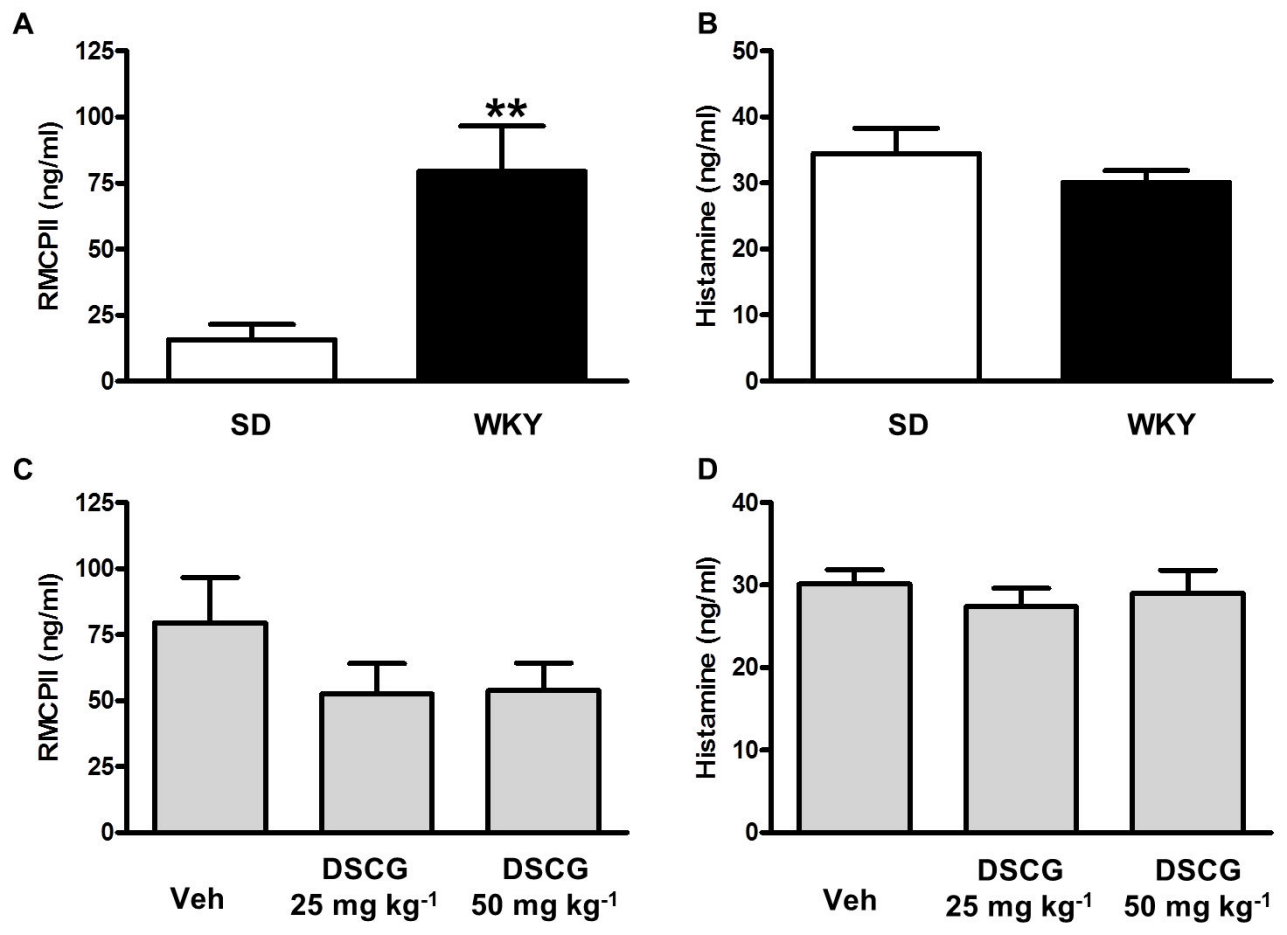
Stimulated release of colonic mast cell mediators was insensitive to inhibition by disodium cromoglycate. Compound 48/80 (10 µg ml^-1^)-stimulated RMCPII release was significantly greater from Wistar Kyoto (WKY) colons relative to Sprague Dawley (SD; A) tissues and was insensitive to inhibition by DSCG (C). Similarly, histamine release was neither elevated from WKY colon relative to SD tissues (B), nor sensitive to inhibition by DSCG (D). n= 7-8. ** *p* <0.01, *versus* SD.

Vehicle-treated WKY animals displayed significantly greater compound 48/80-stimulated RMCPII release relative to vehicle-treated SD tissues (t(13)=3.33, *p*<0.01; Figure 2A). However, this was not the case for histamine (Figure 2B), and neither RMCPII nor histamine release were significantly different between WKY and SD tissues following pre-treatment with either dose of DSCG (Figure 2C and D). 

### Disodium cromoglycate inhibits both compound 48/80-stimulated and forskolin (cAMP)-stimulated electrolyte transport in Wistar Kyoto rat colon

**Figure 3:**
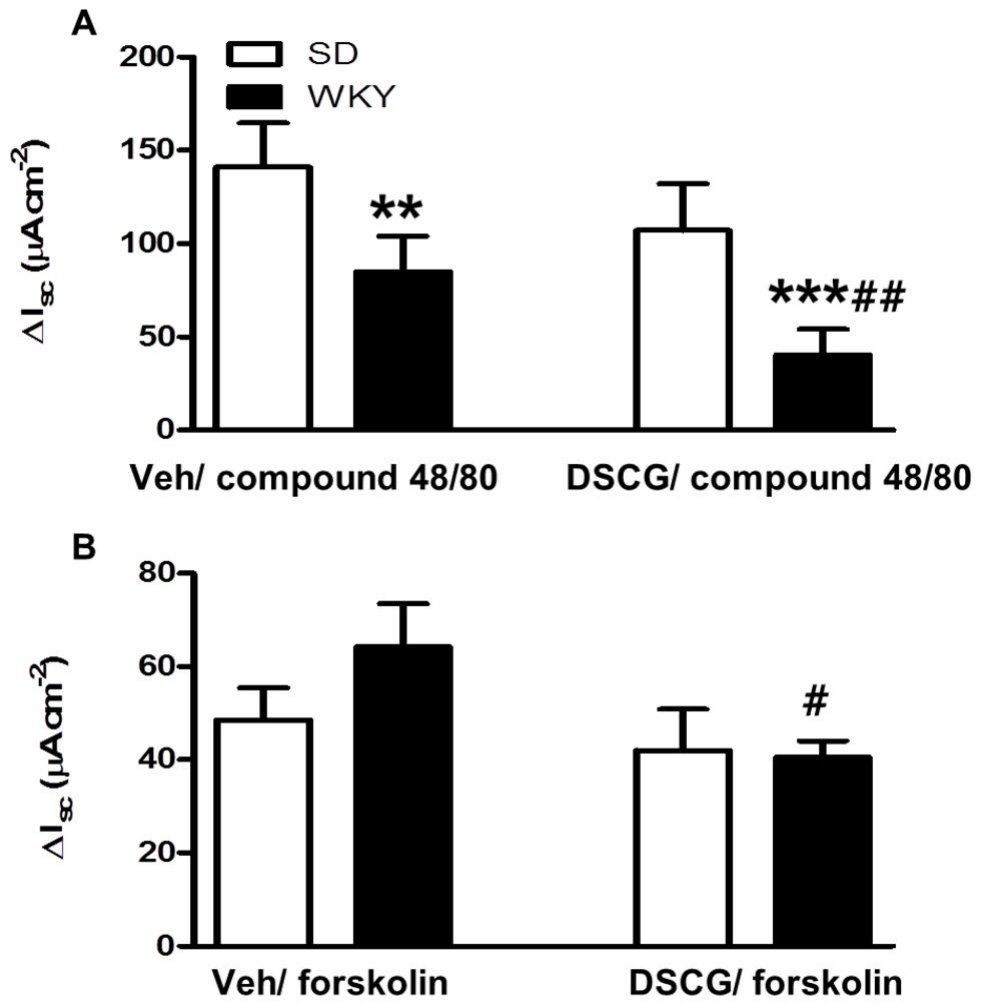
Wistar Kyoto rat colon displays altered sensitivity to compound 48/80 and disodium cromoglycate *in* *vitro*. The change in short-circuit current (Isc) induced by compound 48/80 was significantly decreased in Wistar Kyoto (WKY) colon, and sensitive to inhibition by disodium cromoglycate (DSCG; A). Though WKY and Sprague Dawley tissues responded in a similar manner to forskolin stimulation, only the WKY response was sensitive to inhibition by DSCG. n=7-8, ** *p* <0.01, *** p<0.001, *versus* SD. # *p* <0.05, ## *p* <0.01, *versus* vehicle.

The compound 48/80-induced Isc response was significantly diminished in WKY colon relative to SD tissues (F (1,57),=77.69, *p*<0.001), and this response was sensitive to inhibition by DSCG in WKY tissues only (*p*<0.01; Figure 3A). Similarly, the forskolin-induced increase in Isc was only sensitive to inhibition by DSCG in WKY colon (F(1,56)=6.29, *p*<0.05; Figure 3B).

### Stress-sensitive Wistar Kyoto rats display increased colonic submucosal and intermediate peritoneal mast cells

**Figure 4:**
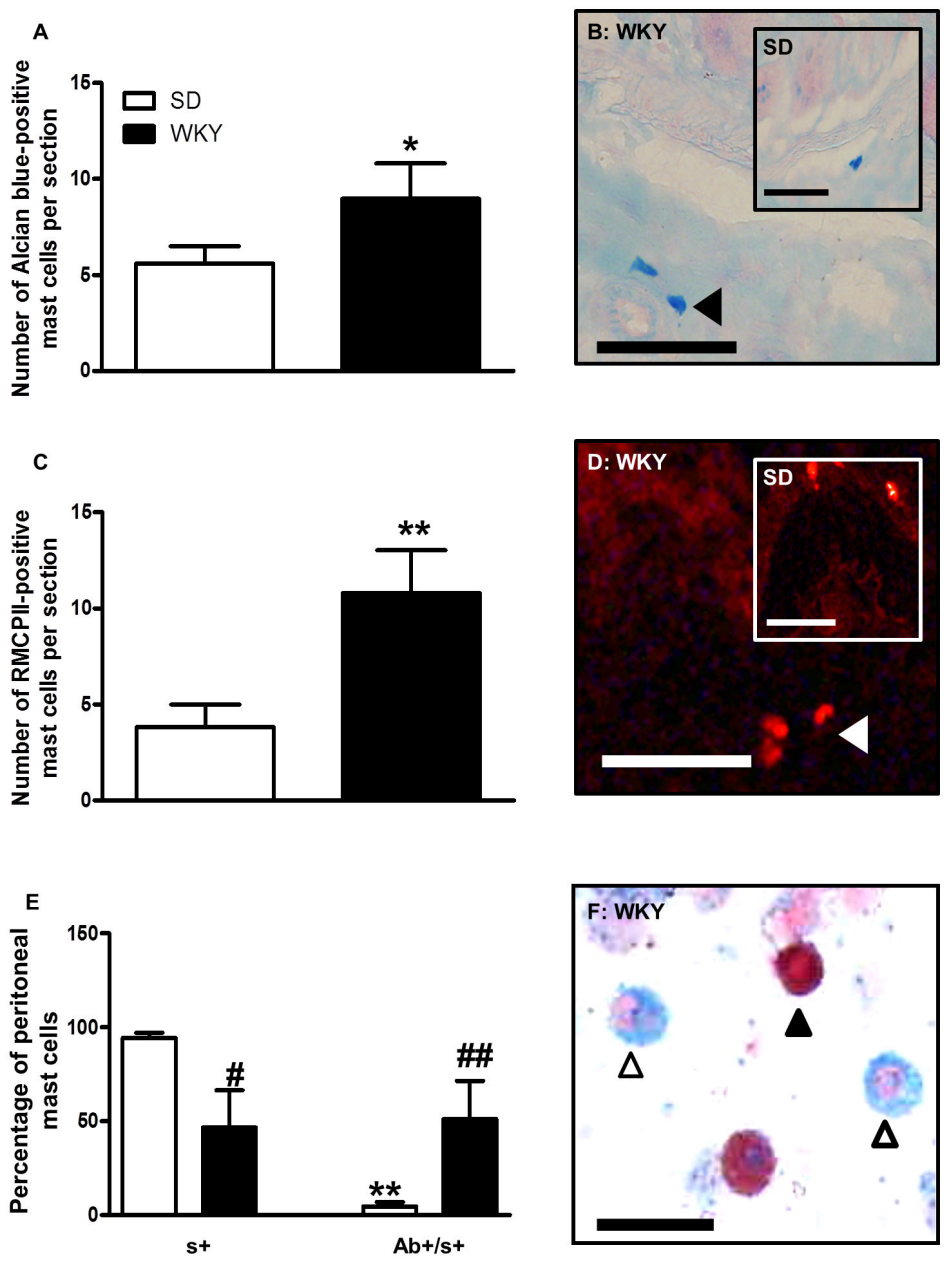
Changes in submucosal mast cell number and peritoneal mast cell classification in Wistar Kyoto rats. Colons from Wistar Kyoto (WKY) animals displayed a significantly greater number of Alcian blue (A and B, black arrow head) and RMCPII (C and D, white arrow head) positive submucosal connective tissue mast cells relative to Sprague Dawley (SD; *insert*) animals (* *p*<0.05, ** *p*<0.01 *versus* SD, n=10-15). Purified peritoneal mast cells isolated from SD animals were predominantly safranin positive (F, black arrow head), and therefore considered CTMC, while equal proportions of those isolated from WKY animals were either safranin positive or Alcian blue/safranin positive (F; white arrow heads), representing CTMC and intermediate mast cells respectively. n=4. * *p*<0.05, versus s^+^. # *p*<0.05, ## *p*<0.01, *versus* SD.

An equal proportion of rat colonic submucosal mast cells may be expected to stain positively for Alcian blue and safranin [33]. However in stress-sensitive WKY animals, submucosal mast cells stained almost exclusively with Alcian blue; significantly more of which were observed in the colonic submucosa of distal sections obtained from WKY animals relative to those from normo-anxious SD rats (t (18)=1.6, *p*<0.05; Figure 4A & B). Moreover, significantly greater numbers of submucosal mast cells expressed RMCPII relative to SD tissues (t (17)=2.7, *p*<0.01; Figure 4 C & D). We found no significant difference in the number of RMCPII-containing MMC between the distal colons of WKY and SD animals (WKY, 53.7 ± 4.0 mast cells/mm^2^ *versus* SD, 48.6 ± 2.6 mast cells/mm^2^, n=10)

To further characterise mast cells in stress-sensitive WKY animals, we isolated peritoneal mast cells from WKY and SD rats. Using a previously described classification method [34,35], our analysis revealed a significant difference between mast cell staining characteristics (F(1,24)=17.84, *p*<0.001) and a significant interaction between rat strain and mast cell staining (F(1,24)=8.18, *p*<0.01). In stress-sensitive WKY rats, approximately 50% of the isolated mast cells displayed classic CTMC staining (safranin positive), significantly less than that observed in SD rats (*p*< 0.05), with the majority of the remaining mast cells displaying an intermediate (Alcian blue/safranin) staining pattern (Figure 4 E & F).

## Discussion

### Disodium cromoglycate reverses visceral hypersensitivity independent of colonic mast cell stabilisation

A heightened response to CRD is a characteristic feature of stress-sensitive WKY animals [5-7,36], and, as mast cells have been implicated in the development of stress-induced visceral hypersensitivity in rats [18,21,37,38], we examined the dose-dependent effect of the mast cells stabiliser, DSCG on visceral hypersensitivity in WKY animals. We specifically selected DSCG based on its proposed selectivity for rat CTMC [26], and on our previous observation of increased colonic CTMC-like mast cells in an early life stress stress-induced model of visceral hypersensitivity [18]. Moreover, DSCG has previously been demonstrated to significantly inhibit nerve injury-induced hyperalgesia in rats [29]. However, to the best of our knowledge, DSCG has not been examined with respect to its effects on abdominal visceral hypersensitivity. In this context, DSCG ameliorated the threshold to first pain behaviour, and total number of pain behaviors, induced by CRD in viscerally hypersensitive WKY rats, at the highest dose tested. However, the inhibition of the visceromotor response to CRD occurs independent of a significant inhibition of colonic RMCPII or histamine release from tissues exposed to DSCG *in vivo*. Despite not reaching statistical significance, we did observe an approximate 30% reduction in colonic RMCPII release from animals treated with 50 mg Kg^-1^DSCG. While we hypothesise that the increased compound 48/80-stimulated RMCPII release in WKY rats may originate from increased submucosal RMCPII-containing mast cells, stabilisation of CTMC-like cells with DSCG may also result in a loss of CTMC-mediated inhibition of MMC mediator release, previously described in rat airways [39]. Moreover, the lack of inhibition observed with respect to stimulation of histamine release by compound 48/80 is in keeping with previous reports on compound 48/80-stimulated release from intestinal mast cells [40]. Collectively, these data point toward a partial inhibition of mast cells by DSCG, but also suggest that the inhibition of visceral hypersensitivity by DSCG may occur independent of colonic mast cell stabilisation. Furthermore, previous studies have demonstrated a significant inhibitory effect of DSCG (10 mg Kg^-1^) on serum histamine levels following IgE-dependant mast cell activation *in vivo* [30]. Therefore, we cannot exclude the possibility that DSCG, in our study, may have stabilised peritoneal mast cells relative to intestinal mast cells, as we did not assess systemic markers of mast cell stabilisation. 

However, DSCG has also been shown to directly influence nociceptive signalling through an inhibition of capsaicin-induced activity of sensory nerve endings [41], and can inhibit substance P binding [42]. The inhibition of visceral hypersensitivity in WKY rats may also reflect a non-specific analgesic effect of the mast cell stabiliser, particularly as we did not assess the effects of DSCG in SD rats. However, Zuo et al., (2003) observed no significant effect of DSCG on hind paw responses to mechanical or thermal hyperalgesia when they examined the contralateral side of animals who had undergone partial ligation of the left sciatic nerve [29]. Moreover, DSCG can inhibit enteric nerve excitation [43] and intestinal contractility [44], both of which may influence the response to CRD. Intriguingly however, DSCG, at the same dose as that used in our study (50 mg Kg^-1^; i.p.), significantly improved anxiety-like behaviours in rats exposed to acute restraint stress [45]. Though we did not assess anxiety-like behaviours in our animals following treatment with DSCG, the anxiolytic properties of the drug may have influenced the response to CRD in WKY rats. 

Therefore, the mechanism by which DSCG reverses the visceral pain response in stress-sensitive animals remains unclear, but is likely to involve several mechanisms, not only limited to mast cells, but also including direct effects on visceral nociceptors, intestinal physiology and centrally-mediated behaviour. 

### Influence of compound 48/80 and disodium cromoglycate on colonic short-circuit current

Despite the absence of a significant inhibitory effect of DSCG on colonic mast cell mediator release from WKY tissues, compound 48/80-stimulated RMCPII release was significantly increased relative to SD colon. As previous studies have demonstrated an influence of RMCPII on intestinal ion transport [46], and given we observed CTMC-like mast cells in the submucosa of WKY rat colon, we examined the effects of the CTMC activator, compound 48/80 and CTMC inhibitor, DSCG on colonic ion transport in submucosal-mucosal colonic preparations. Previous studies, albeit in chicken ileal preparations, previously characterised the compound 48/80-induced change Isc to be histaminergic and sensitive to inhibition with the mast cell stabiliser, ketotifen, and the histamine receptor 1 antagonist, mepyramine [47]. Despite observing an increased number of submucosal mast cells in WKY tissues, the compound 48/80 response was significantly decreased in WKY colon relative to that observed in tissues from normo-anxious SD animals. However, more recent data have identified a mast cell independent, direct effect, of compound 48/80 on submucosal neurones [43]. Therefore, our data may indicate a change in enteric nervous system neuroplasticity or in the neurochemistry of submucosal neurones in WKY rat colon. Moreover, DSCG appears to exert a non-selective inhibitory effect on enteric nerve excitation [43]. Therefore, further investigation is warranted into teasing apart the mast cell- and neuronal-components of the compound 40/80-induced change in Isc in rat colon. Nontheless, only WKY tissues displayed altered sensitivity to both compounds. 

The mechanism for this differential sensitivity to DSCG between SD and WKY rats remains uninvestigated, but may be due to strain-related differences in the response to compound 48/80 [48]. Moreover, the inhibitory effects of DSCG can be enhanced by additional factors, for example alterations in cAMP or sympathetic activity [49], which may be relevant in our study, given WKY tissues displayed an increased Isc response to the cAMP activator, forskolin, though this did not reach significance. Moreover, others have demonstrated an inhibition of corticotrophin releasing factor-induced Isc by the mast cell stabiliser, doxantrazole [50], receptors for which our group previously found to be altered in the colon of WKY animals [51]. Therefore, these data suggest that locally, in the colon, and in a strain dependant manner, DSCG can inhibit either mast cell- or neurally-stimulated Isc. And although forskolin-stimulated Isc was reduced in WKY tissues in the presence of DSCG, the resultant Isc was no different to that observed in control, SD tissues. 

### Characterisation of colonic and peritoneal mast cells from stress-sensitive rats

In stress-sensitive WKY animals we observed an increase in colonic submucosal mast cells, similar to our previous findings in a rat model of early life stress [18]. In particular, these mast cells were Alcian blue positive, with few if any safranin positive mast cells observed, and they displayed characteristics of CTMC, such as resistance to paraformaldehyde fixation [52]. Our findings are in contrast to previous studies, in which equal proportions of Alcian blue and safranin positive mast cells were observed in the rat colonic submucosa [33]. Therefore, we speculate that the Alcian blue positive mast cells observed in the WKY colonic submucosa most likely reflect a population rich in chondroitin sulphate and low in heparin [53]. 

In contrast to the colon, a significant proportion of peritoneal mast cells obtained from WKY rats displayed Alcian blue/safranin staining characteristics with a concomitant, and significant, decrease in safranin CTMC relative to control SD rats; in which the majority of peritoneal mast cells were safranin positive, a classic feature of CTMC [54,55]. These Alcian blue/safranin intermediate mast cells may not only reflect an immature mast cell population [35], but also a population in a trans-differentiated state, the process whereby mature mast cells change the content of their cytoplasmic granules [56,57] or reversibly acquire features of immature mast cells in response to the local microenvironment [58,59]. Intermediate mast cells may also represent increased mast cell turnover [60]. However, the microenvironmental factors influencing the characteristics of colonic and peritoneal mast cells in WKY rats remains unexplored, and likely include a variety of factors including cytokines, neurotransmitters and lipid-derived mediators among others, which are also most likely to vary depending on their tissue localisation. Nonetheless, we have observed alterations in both the number (colon) and classification (peritoneal) of mast cells in stress-sensitive rats, and we speculate that such alterations may be associated with the anxiety-like phenotype of WKY rats given the evidence that inhibition of mast cells, with DSCG, influences anxiety-like behaviours *in vivo* [45,61]. 

### Concluding remarks

We have demonstrated that DSCG displays efficacy in reducing abdominal visceral pain, though the precise mechanism is likely multi-faceted, and not solely due to colonic mast cell stabilisation. Moreover, we identified that compound 48/80-induced responses in the colon of WKY animals were sensitive to inhibition with DSCG, and this may account for the symptom improvement (bowel movement and stool consistency) observed in diarrhoea-predominant IBS patients treated with the drug [62].We have further shown that mast cell number and classification, in the colon and peritoneum, are increased and altered respectively in a stress-sensitive animal model of visceral hypersensitivity. This coupled with our previous work on the impact of early life stress on mast cell phenotype, and the development of visceral hypersensitivity [18], highlights a role not just for mast cells in the pathogenesis of stress-related GI disorders, but also their heterogeneity. However, alterations in mast cells are likely to occur downstream of local microenvironmental influences, investigations of such milieu is now warranted. 
